# Evaluating progress towards triple elimination of mother-to-child transmission of HIV, syphilis and hepatitis B in the Netherlands

**DOI:** 10.1186/s12889-019-6668-6

**Published:** 2019-03-29

**Authors:** Maartje Visser, Catharina P. B. van der Ploeg, Colette Smit, Chantal W. P. M. Hukkelhoven, Frithjofna Abbink, Birgit H. B. van Benthem, Eline L. M. Op de Coul

**Affiliations:** 1 National Institute for Public Health and the Environment, Epidemiology and Surveillance unit, P.O. Box 1, 3720 BA Bilthoven, the Netherlands; 2 Netherlands Organisation for Applied Scientific Research TNO, Schipholweg 77-89, 2316 ZL, Leiden, The Netherlands; 3 HIV Monitoring Foundation, Tafelbergweg 51, 1105 BD Amsterdam, the Netherlands; 4 Perined, Mercatorlaan 1200, 3528, BL Utrecht, the Netherlands; 5 National Institute for Public Health and the Environment, Centre for population screening, P.O. Box 1, 3720 BA Bilthoven, the Netherlands

**Keywords:** Pregnant women, Antenatal screening, HIV, Congenital syphilis, Hepatitis B

## Abstract

**Background:**

In 2014 the World Health Organisation (WHO) established validation criteria for elimination of mother-to-child transmission (EMTCT) of HIV and syphilis. Additionally, the WHO set targets to eliminate hepatitis, including hepatitis B (HBV). We evaluated to what extent the Netherlands has achieved the combined WHO criteria for EMTCT of HIV, syphilis and HBV.

**Methods:**

Data of HIV, syphilis and HBV infections among pregnant women and children (born in the Netherlands with congenital infection) for 2009–2015, and data required to validate the WHO criteria were collected from multiple sources: the antenatal screening registry, the HIV monitoring foundation database, the Perinatal Registry of the Netherlands, the national reference laboratory for congenital syphilis, and national HBV notification data.

**Results:**

Screening coverage among pregnant women was > 99% for all years, and prevalence of HIV, syphilis and HBV was very low. In 2015, prevalence of HIV, syphilis and HBV was 0.06, 0.06 and 0.29%, respectively. No infections among children born in the Netherlands were reported in 2015 for all three diseases, and in previous years only sporadic cases were observed In 2015, treatment of HIV positive pregnant women was 100% and HBV vaccination of children from HBV positive mothers was > 99%. For syphilis, comprehensive data was lacking to validate WHO criteria.

**Conclusions:**

In the Netherlands, prevalence of maternal HIV, syphilis and HBV is low and congenital infections are extremely rare. All minimum WHO criteria for validation of EMTCT are met for HIV and HBV, but for syphilis more data are needed to prove elimination.

## Background

In the Netherlands, all pregnant women are offered screening for Human Immunodeficiency Virus (HIV), syphilis and hepatitis B virus (HBV). Participation, including blood sample collection, is according to the opting-out-principle during the first midwife appointment in the first trimester of pregnancy. In case of positive test results, confirmatory tests are performed, and in case of confirmed positive test results women are referred to secondary care for treatment and/or other preventive measures [1]. In the Netherlands, standard care for sexually transmitted infections includes partner services but execution and data collection of testing and treatment for partners is not embedded in the screening programme. In 2011, an evaluation of the Dutch antenatal screening programme for HIV, HBV and syphilis was published for the years 2006–2008, which concluded that the programme is effective in detecting HIV, HBV and syphilis among pregnant women and in preventing vertical transmission [2]. It was estimated that 5–10 HIV, 10 syphilis and 50–75 HBV cases in newborns had been prevented annually due to screening [2].

In 2014, the World Health Organisation (WHO) established a list of validation criteria to facilitate efforts of elimination of mother-to-child transmission (EMTCT) of HIV and syphilis, which were updated in 2017 [3]. These criteria consist of a global minimum of three impact- and six process indicators, of which an overview is given in Table 1. The WHO has not yet established elaborate guidance on EMTCT of HBV, but it is addressed in the WHO Global health sector strategy on viral hepatitis 2016–2021 [4]. It states that prevention of MTCT is a core intervention area in ending the hepatitis epidemic, mainly through timely HBV birth-dose vaccination, antenatal testing, and the use of antiviral drugs. In this global strategy, the WHO also set targets to reach EMTCT of HBV in 2030. These additional targets are also included in Table 1.

**Table 1:** Minimum required WHO indicator criteria for validation of EMTCT of HIV and syphilis, and additional criteria for hepatitis B

Indicators		Netherlands 2014	Netherlands 2015	Criterion met?
Shared indicators HIV and syphilis				
ANC coverage (at least one visit) of ≥95%		99.2%^a^	99.7%^a^	Yes
HIV indicators				
Coverage of pregnant women who know their HIV status of ≥95%		99.2%^a^	99.7%^a^	Yes
Antiretroviral (ARV) coverage of HIV positive pregnant women of ≥95% (women with suppressed viral load at delivery)		96% (84%)^b^	100% (97%)^b^	Yes
Case rate of new paediatric HIV infection due to mother-to-child transmission (MTCT) of HIV of ≤50 cases per 100,000 live births		0.57^b^	0.00^b^	Yes
MTCT rate of HIV of < 5% in breastfeeding population OR MTCT rate of < 2% in non-breastfeeding populations		0.75%^b^		Yes
Syphilis indicators				
Coverage of syphilis testing of pregnant women of ≥95%		99.2%^a^	99.7%^a^	Yes
Treatment of syphilis-seropositive pregnant women ≥95% (% registered referred to care)		72.6%^a^	70.5%^a^	Unknown
Case rate of congenital syphilis ≤50 cases per 100,000 live births		0.00^c^-1.14^d^	0.00^c^-0.59^d^	Presumably
Hepatitis B indicators				
Hepatitis B virus vaccination: childhood vaccine coverage (third dose coverage) ≥90%		93.1%^e^	92.2%^e^	Yes
Hepatitis B virus birth-dose vaccination coverage or other approach to prevent mother-to-child transmission ≥90%	Birth-dose vaccination	99.4%^e^	99.4%^e^	Yes
	HBIg admission at birth	99.8%^f^	99.8%^a^	
HBsAg prevalence among children ≤0.1%		0.0%^g^	0.0%^g^	Yes

Sources

^a^Antenatal screening registry (Praeventis) data, PSIE procesmonitor 2015 [5]

^b^ATHENA database (Stichting HIV Monitoring)

^c^RIVM-CIb-IDS laboratory data

^d^Perinatal registry data (Perined)

^e^national immunisation programma data, RIVM report 2018-0008 [7]

^f^Antenatal screening registry (Praeventis) data, PSIE procesmonitor 2014 [18]

^g^Osiris database of notifiable diseases

HIV, syphilis and hepatitis B are low prevalent diseases in the Netherlands; hence, EMTCT is likely to be achievable. To assess whether the Netherlands can indeed meet the WHO criteria for EMTCT of HIV and syphilis, we re-evaluated the Dutch antenatal screening programme on HIV and syphilis for the years 2009–2015. To review EMTCT of HBV we used the additional WHO global strategy criteria. In the current study only the minimum indicators are addressed. To achieve official validation, additional data collection and analyses will have to be described in a full country report, such as extensive case studies, evaluation of additional criteria, and an assessment of data- and laboratory quality. Detailed information can be found in the WHO guidance document [3]. This study can hence be seen as a first exploration of the feasibility of EMTCT, and will provide insight in future efforts that are needed to strengthen EMTCT efforts and surveillance of EMTCT in the Netherlands.

## Methods

Participation data and outcomes of the antenatal screening are registered in an electronic database (Praeventis) in the Netherlands. As every year a process evaluation is performed on these data, information on screening uptake and general screening outcomes have been well-reported in the past [5]. Screening outcomes are reported based on laboratory confirmed positive tests. Screening coverage should be calculated by dividing the number of women screened by the number of pregnancies in a given year. Only an estimate can be provided, as the number of pregnancies in a given year is uncertain. It is estimated by the number of children born alive in the Netherlands half a year later (as reported by the Central Bureau of Statistics (CBS)), assuming that children are born 6 months after screening. As pregnancies may result in multiple births, correction of the number of life births is needed. In the Netherlands, the yearly number of twins and triplets is also registered by the CBS, and results in a correction of minus 1.5–1.7% of the number of children born alive. Another correction is needed for loss of pregnancies. In 2012, we investigated the number of pregnancy losses after the first screening among RhD-negative women, as these women participated to a pilot screening of fetal Rhesus-D-typing in week 27 of pregnancy. Loss of pregnancy was registered in 3.8%. For the denominator of the estimate of screening coverage, the number of children born alive was thus corrected by − 1.5-1.7% and by + 3.8% to get an estimate of the number of pregnancies. The numerator of screening coverage, i.e. the number of women screened in a year, was corrected for double registration of the same pregnancy and for screening of women living abroad, as their children are not in the Dutch CBS statistics of lifeborns. We used additional data from the Praeventis database to describe age and ethnicity of pregnant women with positive test results for HIV, syphilis or HBV. Ethnicity was defined using country of birth of the pregnant women; only first generation migrants were considered non-Dutch. Countries were grouped into regions of origin using the UNSTAT list of geographic regions. In Praeventis, not all additional data needed to evaluate the WHO criteria are available. Therefore, information from other data sources was included, as described below.

### HIV

To gain additional information on pregnancies among HIV positive women in the Netherlands, we obtained data from the AIDS therapy evaluation in the Netherlands (ATHENA) cohort, which is maintained by the ‘Stichting HIV monitoring’ (SHM). The ATHENA national observational HIV cohort includes data from all persons with HIV in care in the Netherlands in any of the 26 HIV treatment centres. Pregnancies among HIV positive women are also registered in ATHENA. To be able to compare annual numbers of HIV positive pregnant women between the ATHENA cohort and Praeventis data (which is based on screening date), we estimated the date of screening for the women in the ATHENA cohort. For women who were newly diagnosed with HIV during pregnancy, we used the date of diagnosis as the date of screening. For the other women, we used the date of their 12th week of pregnancy, derived from the estimated due date, as this is the average moment of screening. If the estimated due date was not known, which was mostly the case for pregnancies that did not result in delivery, we used the date of termination of the pregnancy.

Data on children with congenital HIV infection were also obtained from the ATHENA database. We included children who were infected through vertical transmission and who were born in the Netherlands in 2009–2015. To calculate the MTCT rate of HIV, we used the number of children born with HIV divided by the number of registered births given by HIV positive women from the ATHENA database.

### Syphilis

To gain insight in the number of children born with syphilis, two alternative data sources were used. First, we used data from the CIb-IDS (Centre for Infectious diseases research, Diagnostics and Screening) laboratory at the National Institute for Public Health and the Environment (RIVM), where immunoglobulin M diagnostics are performed on children aged < 1 years who are suspected of having congenital syphilis. Second, we acquired data for 2009–2015 from the Perinatal Registry of the Netherlands (Perined), which includes congenital syphilis diagnoses reported by paediatricians. Perined contains – among others – diagnoses from all registered children admitted to a paediatric ward within 28 days after birth. In 2015, 86% of Dutch paediatric practices reported data to Perined [6].

The WHO has established a global surveillance case definition for congenital syphilis, to be used with the validation criteria: 1) A stillbirth, live birth, or foetal loss at > 20 weeks of gestation or > 500 g to a syphilis-seropositive mother without adequate syphilis treatment, or: 2) a live birth, stillbirth or child aged < 2 years born to a woman with positive syphilis serology or with unknown serostatus, and with laboratory and/or radiographic and/or clinical evidence of syphilis infection (regardless of the timing or adequacy of maternal treatment) [3]. While there is registration of pregnancy outcomes in Perined, cause of death is not registered for stillbirths in the Netherlands, nor is there a comprehensive registration of syphilis serostatus of the mother in case of a stillbirth. Therefore, we do not have insight in congenital syphilis resulting in stillbirths, and are not able to follow the exact WHO case definition. In this study, all registered cases of congenital syphilis include only live born children.

### Hepatitis B

Acute and chronic HBV infections are notifiable in the Netherlands and are registered in the National Register for Notifiable Diseases (Osiris) at the RIVM. Reason for testing is not well registered in this database, so we could not distinguish newly notified infections from pregnant women. To obtain information on HBV infections among children, we collected data on children aged < 2 years old who were born in the Netherlands and for whom vertical transmission was specified as the most likely route of transmission from the Osiris database. Data on HBV vaccination coverage are reported in the annual RIVM report on the national immunisation programme [7] and in the prenatal screening process evaluation [5].

## Results

### Screening coverage

The estimated coverage of the antenatal screening programme was > 99% in all years from 2009 to 2015. In 2015, 83.3% of women were tested before the 13th week of pregnancy. Of the 175,933 screened women, 3 women refused syphilis testing and 6 women refused HBV testing. One hundred twenty-nine women refused HIV testing, which was 0.07% of the total population of pregnant women [5]. In 2014, test refusal occurred 5 times for HBV and syphilis and 87 times for HIV. Hence, the WHO criteria of antenatal care coverage, HIV testing and syphilis testing for ≥95% of pregnant women were all met (Table 1).

### HIV

**Fig. 1:**
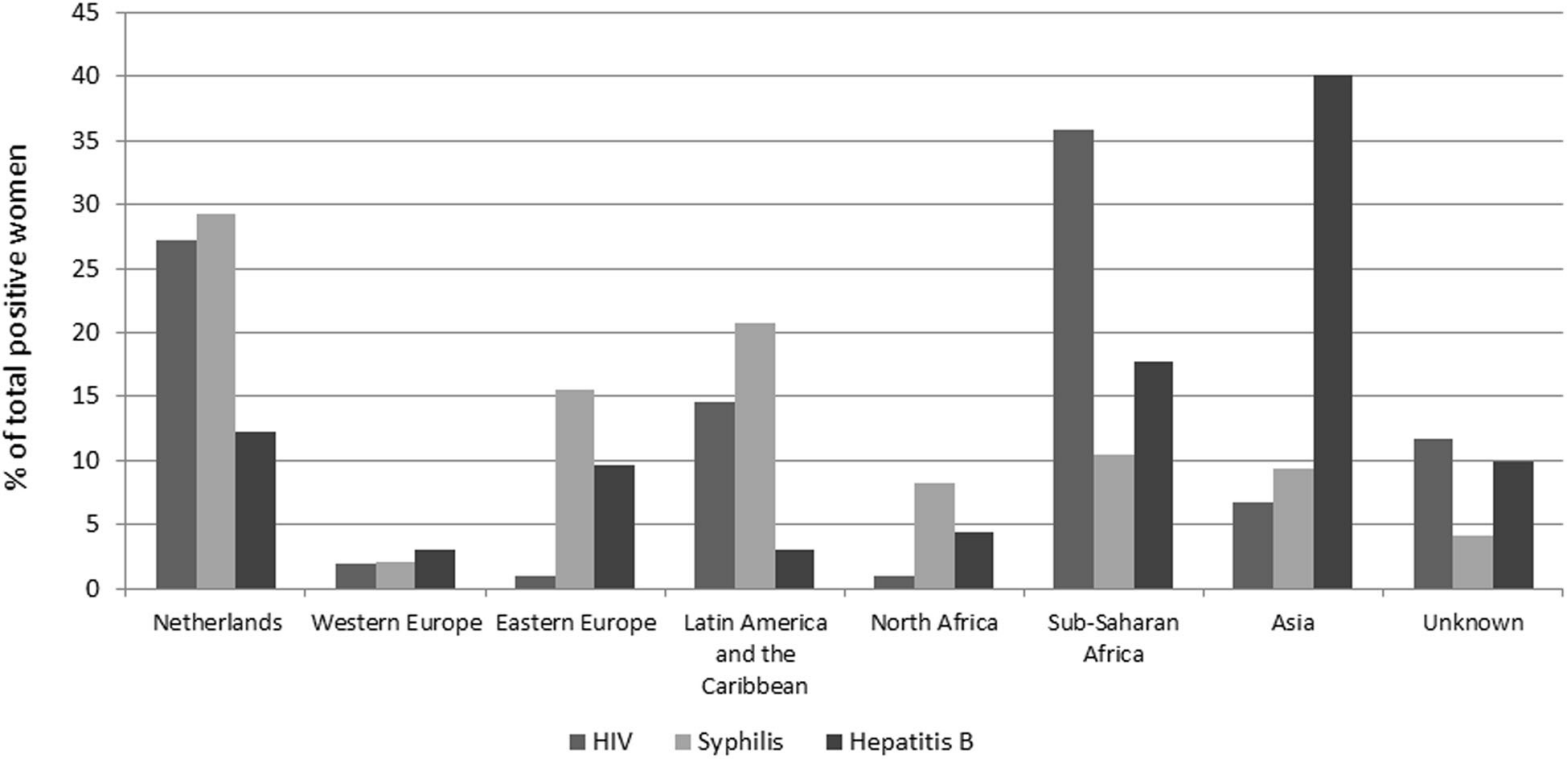
Ethnicity of women who tested positive for HIV, syphilis or hepatitis B in antenatal screening in the Netherlands, 2015 (source: Praeventis database)

Table 2 shows the number of HIV cases among pregnant women in the Praeventis and ATHENA databases. Between 99 and 113 women were found positive by screening between 2012 and 2015, corresponding with an annual estimated HIV prevalence of 0.06% [5]. The median age of HIV positive women in Praeventis (in 2015) was 32 (interquartile range [IQR] 29–37). Of the 103 women with a positive HIV test result in 2015, 36% originated from Sub-Sahara Africa, 27% from the Netherlands, and 15% from Latin America or the Caribbean (Fig. 1). The numbers of pregnant women with HIV in the Praeventis and ATHENA databases did not fully correspond. The numbers of pregnancies among HIV positive women in the ATHENA database show a decreasing trend. Between 2009 and 2015, on average 13.5% of pregnancies registered at ATHENA concerned women who were newly diagnosed with HIV during that pregnancy (Table 2). It is therefore likely that most women who tested positive in screening (Praeventis) were already familiar with their HIV infection. According to data registered in Praeventis, in 2015 at least 21% of HIV positive pregnant women were newly diagnosed through screening. For 66% the infection was known before screening and for 14% this was unknown [5].

**Table 2:** Numbers of registered HIV, syphilis and hepatitis B infections among pregnant women in the Netherlands, 2009–2015

	HIV				Syphilis		Hepatitis B	
	Praeventis		ATHENA		Praeventis		Praeventis	
Year	Total no. of infections	Estimated prevalence	Total no. of infections	% Newly diagnosed	Total no. of infections	Estimated prevalence	Total no. of infections	Estimated prevalence
2009	-	-	151	14.6	-	-	-	-
2010	-	-	167	13.8	-	-	-	-
2011	-	-	149	18.1	-	-	-	-
2012	113	0.07%	138	10.1	101	0.06%	536	0.31%
2013	99	0.06%	111	9.9	135	0.08%	529	0.30%
2014	100	0.06%	86	8.1	97	0.06%	559	0.32%
2015	105	0.06%	70^a^	20.0	98	0.06%	506	0.29%

^a^2015 could be incomplete due to delay in registration

- = not shown due to changes in registration (year classification from June–June to January–January)

#### WHO criteria for HIV

The first HIV WHO criterion calls for ≥95% antiretroviral therapy (cART) coverage among pregnant women. In ATHENA, cART use during pregnancy in 2014 was 89% for the total number of pregnant women, and 96% for women with a pregnancy resulting in delivery. In 2015, 96%, respectively 100% were treated with cART. Furthermore, 84% of pregnant women in 2014 had a suppressed viral load at time of delivery (HIV RNA < 50 copies/mL), which increased to 97% in 2015. Hence, not only the number of women on cART was above the WHO criterion of 95% in 2015, but also the number of women with a suppressed viral load at time of delivery (Table 1). The increase is likely due to the implementation of universal HIV treatment in 2015.

The second WHO criterion is a case rate of paediatric HIV infections due to MTCT of ≤50 cases per 100,000 live births. Between 2009 and 2015, yearly between 0 and 3 HIV positive children born in the Netherlands were registered in ATHENA (Table 3), resulting in an annual case rate of < 2 per 100,000 live births for all years, which is well below the WHO criterion (Table 1).

**Table 3:** Numbers of registered congenital infections among children, and case rate per 100,000 live births for HIV, hepatitis B and syphilis in the Netherlands, 2009–2015

		HIV^b^		Hepatitis B^c^		Syphilis^d^	
Birth year	Total no. of live births^a^	no. of infections	Case rate per 100,000 live births	no. of infections	Case rate per 100,000 live births	no. of infections	Case rate per 100,000 live births
2009	184,397	0	0.00	2	1.08	0–2	0.00–1.08
2010	184,397	3	1.63	2	1.08	1–6	0.54–3.25
2011	180,060	0	0.00	1	0.56	0–6	0.00–3.33
2012	175,959	1	0.57	1	0.57	1–2	0.57–1.14
2013	171,341	1	0.58	0	0.00	1–3	0.58–1.75
2014	175,181	1	0.57	0	0.00	0–2	0.00–1.14
2015	170,510	0	0.00	0	0.00	0–1	0.00–0.59

^a^Source: Statistics Netherlands

^b^Source: ATHENA database (Stichting HIV Monitoring)

^c^Source: Osiris database of notifiable diseases

^d^Source: RIVM-CIb-IDS laboratory (left) and perinatal registry (Perined) (right)

The third HIV WHO criterion is an MTCT rate of HIV of < 5% in breastfeeding populations, and < 2% in non-breastfeeding populations. To calculate the MTCT rate for HIV, we divided the number of HIV infected children by the number of pregnancies with delivery in ATHENA. As the Netherlands is a low prevalence country for HIV, we pooled 2014 and 2015 data, as suggested in the WHO guidelines. For 2014 and 2015 combined, the MTCT rate was 0.75% (1/133), which is well below the WHO criterion (Table 1). One child born with HIV in 2014 had a mother who became infected after the antenatal screening.

### Syphilis

In Praeventis, 101 (2012), 135 (2013), 97 (2014) and 98 (2015) women were registered as syphilis positive, resulting in an estimated prevalence of 0.06–0.08% among pregnant women in the Netherlands (Table 2). Median age of syphilis-positive women in 2015 was 33 (IQR 29–37.5). Most women were of non-Western origin (64.5% in 2015), mainly including Eastern Europe, Latin America, and the Caribbean (Surinam and the former Dutch Antilles) (Fig. 1). It should be noted that the number of syphilis positive women registered in Praeventis is subjected to some uncertainty due to diagnostic procedures. Laboratory results were directly sent to Praeventis, and hence did not reflect a diagnosis of syphilis but merely a positive test. Because in screening higher VDRL cut-offs are used (1:8 instead of 1:4 or 1:2) to decide whether a positive test is clinically relevant, some of these women tested positive but did not have an active syphilis infection. These positive tests are indicating infections that were already treated or spontaneously resolved, or caused by endemic treponematoses, and thus do not require actions to be taken. Expectations are that the true number of pregnant women with active/clinically relevant syphilis infections is much lower. In 2016, the laboratory results reported with screening outcome were evaluated more strictly by the RIVM, resulting in only 36 women being reported as syphilis positive after screening [8].

#### WHO criteria for syphilis

The first WHO criterion for syphilis regards treatment of syphilis-positive women. A referral to secondary care was registered for 70.5% of syphilis positive pregnant in Praeventis in 2015 [5] (Table 1). It is however likely that the percentage of women in care is higher in reality due to missing information in the registration. On the other hand, women registered as syphilis-positive but without active infection do not require treatment, which could explain the low percentage. We expect that the WHO criterion of treatment of syphilis-seropositive pregnant women ≥95% is therefore met in reality, but unfortunately current data are incomplete.

The second WHO criterion is a case rate of congenital syphilis of ≤50 cases per 100,000 live births. Among children, the number of reported infections differed between the two used data sources. At the RIVM CIb-IDS laboratory, three children were confirmed positive for syphilis between 2009 and 2015. The Perined registry reported 22 children with congenital syphilis between 2009 and 2015 (Table 3). We therefore calculated a minimum and a maximum case rate for syphilis (Table 3). Both rates are far below the WHO criterion for all years between 2009 and 2015 (Table 1). However, these cases only include live born children, as cause of death for stillbirths is not registered in the Netherlands. Due to the low number of syphilis positive women and the low number of live born children with congenital syphilis, we can safely assume that the number of stillbirth attributable to syphilis would also be extremely low. Therefore, when stillbirths would be included, the case rate would certainly still not exceed 50/100,000 live births.

### Hepatitis B

At the prenatal screening in 2015, 506 women were tested positive for HBV. HBV prevalence was stably low between 0.32 and 0.29% between 2012 and 2015 (Table 2). Most women who tested positive for HBV in the antenatal screening in 2015 were of non-Dutch descent (74.8%). The most reported regions of origin were Asia (40.1%) and Sub-Saharan Africa (17.7%) (Fig. 1). Median age was 31 (IQR 28–35).

#### WHO criteria for hepatitis B

The first WHO criterion for HBV concerns child vaccination. In 2011, universal HBV vaccination was added to the Dutch national immunisation programme. Vaccination coverage is high: for the birth cohort of 2014, 93.1% was fully vaccinated for HBV at age 2, which complies with the WHO indicator of childhood vaccine coverage being ≥90% (Table 1). Vaccination coverage of children from HBV carrying mothers was even higher, with 98.8% receiving their first vaccination within 3 days after birth, 99.2% within 41 days after birth, and 98.4% being fully vaccinated at age 2 [9]. Children from HBV positive mothers also should receive hepatitis B immunoglobulin (HBIg) directly after birth; 99.8% in 2015. For 99.1% of HBIg-administrations the date of administration was known. Among these children, 92.7% received HBIg at the day of birth, and 99.6% within 48 h [5]. Hence, also the second WHO indicator for HBV (HBV virus birth-dose vaccination coverage or other approach to prevent mother-to-child transmission ≥90%) is met (Table 1).

The third WHO HBV criterion is Hepatitis B surface Antigen (HBsAg) prevalence among children of ≤0.1%. Between 2009 and 2012, 6 children were born in the Netherlands with HBV (Table 3). After 2012, no more cases among children < 2 years old have been reported in the Osiris database. Therefore the criterion of HBsAg prevalence ≤0.1% among children is also met.

## Discussion

The Netherlands meets all of the minimum WHO criteria for EMTCT of HIV and HBV. For syphilis, one of the three criteria was met. The prevalence of HIV, syphilis and HBV is very low and stable among pregnant women, and only very few children with congenital infections have been born in the Netherlands in the past years. In 2015 there were 0 reported cases for all three infections.

The estimated coverage of the antenatal screening was very high, with > 99% of pregnant women screened each year. We therefore have reliable information on the prevalence of HIV, syphilis and HBV among pregnant women in the Netherlands. The use of multiple additional data sources enabled us to gain more insight into infections among both pregnant women and children born in the Netherlands, and allowed for assessment of almost all of the WHO EMTCT criteria. However, there are some limitations to address.

First, comparison of the different data sources could only be done on group level instead of individual level. Privacy regulations did not allow for linkage of records across different databases. Therefore we could not gain detailed insight into the overlap and completeness of the different data sources. We could not distinguish between women who were familiar with HIV/HBV infection and women newly diagnosed during screening, and had therefore limited information about the women that were (or were not) linked to care. Second, no data were available of women or children who were not in care. This could have caused a slight underestimation of disease prevalence and/or incidence. Last, there was no information available on voluntary abortions in most included data sources. For the screening coverage calculations, we do not expect voluntary abortions to be of significant influence. Especially since most abortions occur before screening.

The main goal of this study was to assess whether the Netherlands meets the WHO criteria for EMTCT of HIV, syphilis and HBV. For HIV, all data needed to evaluate the WHO criteria was available and of good quality. We can therefore be certain that the criteria for HIV are met in the Netherlands. However, we lack insight in the exact proportion of newly diagnosed women at screening, in whether the women refusing HIV testing at screening might be mostly women who are already diagnosed with HIV, and in whether positively screened women are all in care at an HIV treatment centre, as databases could not be linked. As policy in the Netherlands is to guide all newly diagnosed persons with HIV directly to care in one of the 26 officially recognised HIV treatment centres and start immediate treatment [10], we expect this is well organised for pregnant women. This information can provide important insights into the efficiency of the present health care system, and it would contribute to a more precise estimation of the number of pregnant women newly diagnosed with HIV in the Netherlands.

Also for HBV all data needed were available. Vaccination coverage is well-known due to the national vaccination registration. Furthermore, HBV is a notifiable disease in the Netherlands, and therefore all HBV infections among children should be reported. A decrease in yearly HBV infections among children was seen between 2009 and 2015 (from 2 in 2009 to 0 since 2012). This decrease could be due to increased antiretroviral treatment during pregnancy, which is an effective method to prevent breakthrough infections [11], but it could also be a consequence of changes in the child healthcare system. In 2011, the responsibility for serological testing of children born to HBV positive mothers was transferred to the child’s general practitioner, instead of being nationally coordinated. An evaluation showed that the implementation of this process was suboptimal, and that only half of the children born in 2012 from HBV positive mothers who were included in the evaluation received serological testing [12]. This could have caused breakthrough infections to be missed.

For syphilis, validation of WHO criteria was more difficult due to incompleteness of data. Syphilis is not a notifiable disease in the Netherlands. Although the number of syphilis-positive pregnant women could be extracted from Praeventis, this number is subject to uncertainty due to the aforementioned discrepancy in registration of positive syphilis tests versus clinically relevant (active) syphilis infections. This also hampers the collection of information about accurate treatment of pregnant women with an active syphilis infection. Furthermore, reliable data about congenital syphilis infections were lacking. The two data sources that were used reported different numbers of yearly infections. The RIVM CIb-IDS reference laboratory should receive samples from all children with suspected congenital syphilis. However, in Perined there were more cases of congenital syphilis reported than by the CIb-IDS laboratory. It is unknown which number is true, and whether this difference is seen because of registration errors in Perined or because not all cases of suspected congenital syphilis are being forwarded to the CIb-IDS reference laboratory. Finally, no systematic information about cause of death for stillborn children is collected in the Netherlands, causing a lack of insight into the number of pregnancies that do not result in delivery due to syphilis infection resulting in the inability to evaluate the WHO definition of congenital syphilis.

Until now, ten countries have officially validated EMTCT for both HIV and syphilis. Furthermore, EMTCT of HIV has been reached in Armenia and EMTCT of syphilis was validated in Moldova [3]. No Western-European countries have thus far officially validated EMTCT of HIV, syphilis and/or HBV. However, two evaluation studies were performed in the United Kingdom, which show that the UK meet the minimum WHO criteria for syphilis [13], and that HIV MTCT rates are also very low [14]. Even though in most European countries prevalence of HIV, syphilis and HBV will be relatively low among pregnant women and children, averting also the last cases of vertical transmission is very important.

In case the Netherlands decides to apply for official validation, a validation committee and -team will have to be established, and additional indicators will have to be evaluated such as more in-depth assessments of data and laboratory quality. Furthermore, the WHO recommends for countries with low HIV and syphilis prevalence (such as the Netherlands) to include extensive case studies in the validation report. We believe it will be possible to collect more in-depth data needed for validation in the Netherlands, but this will require great efforts and extensive collaborations between (local) governments, research (and surveillance) organisations, health-care providers and laboratories. Given that an official validation process would therefore be very costly, it is the question whether a low-prevalence country such as the Netherlands should engage in it. On the other hand, evaluating the screening programme and its outcomes in light of the WHO criteria provided us with new recommendations for improvement, mainly concerning limitations in data availability and the surveillance of syphilis. In a progress evaluation, the WHO acknowledges that in most countries case reporting and surveillance of (congenital) syphilis (particularly stillbirths) are less developed than that for HIV [15], which is also the case in the Netherlands. Improvements need to be made especially to the current congenital syphilis surveillance, as at this moment there is no insight in the exact number of children with congenital syphilis per year. The United States and the United Kingdom have reported recent increases in the number of congenital syphilis, indicating that also in developed countries surveillance and EMTCT efforts remain a necessity [16, 17]. Validation of EMTCT of HIV, syphilis and hepatitis B in the Netherlands might thus seem a disproportionate exercise, but this first explorative evaluation showed that even in a low-prevalence country with an extensive screening programme there are still improvements to be made.

## Conclusions

We can conclude that the Netherlands has a well-established antenatal screening programme with high coverage, and that additional measures to reduce mother-to-child transmission of HIV and HBV, such as treatment and vaccination, are well in place. Also the numbers of both maternal and congenital infections of HIV, syphilis and HBV are low. All minimum WHO criteria for validation of EMTCT are met for HIV and HBV but, especially for syphilis, more data are needed to be able to officially validate elimination.

## Abbreviations

ATHENA: Aids Therapy Evaluation in the Netherlands
cART: Combination Antiretroviral Therapy
CBS: Central Bureau of Statistics (Statistics Netherlands)
CIb-IDS: Centre for Infectious Diseases Research, Diagnostics and Screening
EMTCT: Elimination of Mother-To-Child Transmission
HBIg: Hepatitis B Immunoglobulin
HBsAg: Hepatitis B Surface Antigen
HBV: Hepatitis B Virus
HIV: Human immunodeficiency Virus
Perined: Perinatal Registry of the Netherlands
WHO: World Health Organisation
